# Years of life lived with disease and years of potential life lost in children who die of cancer in the United States, 2009

**DOI:** 10.1002/cam4.410

**Published:** 2015-01-27

**Authors:** Peter M de Blank, Quinn T Ostrom, Chaturia Rouse, Yingli Wolinsky, Carol Kruchko, Joanne Salcido, Jill S Barnholtz-Sloan

**Affiliations:** 1 Department of Pediatric Hematology-Oncology, Rainbow Babies and Children's Hospital11100 Euclid Ave, Cleveland, Ohio; 2 Case Comprehensive Cancer Center, Case Western Reserve University School of Medicine11100 Euclid Ave, Cleveland, Ohio; 3 Central Brain Tumor Registry of the United States (CBTRUS)244 East Ogden Ave Suite 116, Hinsdale, Illinois; 4 Department of Physiology & Biophysics, Case Western Reserve University School of Medicine11100 Euclid Ave, Cleveland, Ohio; 5 Pediatric Brain Tumor Foundation of the United States (PBTFUS)302 Ridgefield Ct., Asheville, North Carolina

**Keywords:** Central nervous system, childhood cancer, leukemia, years of life lived with disease, years of potential life lost

## Abstract

Incidence and survival rates are commonly reported statistics, but these may fail to capture the full impact of childhood cancers. We describe the *years of potential life lost* (YPLL) and *years of life lived with disease* (YLLD) in children and adolescents who died of cancer in the United States to estimate the impact of childhood cancer in the United States in 2009. We examined mortality data in 2009 among children and adolescents <20 years old in both the National Vital Statistics System (NVSS) and the Surveillance, Epidemiology, and End Results (SEER) datasets. YPLL and YLLD were calculated for all deaths due to cancer. Histology-specific YPLL and YLLD of central nervous system (CNS) tumors, leukemia, and lymphoma were estimated using SEER. There were 2233 deaths and 153,390.4 YPLL due to neoplasm in 2009. CNS tumors were the largest cause of YPLL (31%) among deaths due to cancer and were the cause of 1.4% of YPLL due to all causes. For specific histologies, the greatest mean YPLL per death was due to atypical teratoid/rhabdoid tumor (78.0 years lost). The histology with the highest mean YLLD per death in children and adolescents who died of cancer was primitive neuroectodermal tumor (4.6 years lived). CNS tumors are the most common solid malignancy in individuals <20 years old and have the highest YPLL cost of all cancers. This offers the first histology-specific description of YPLL in children and adolescents and proposes a new measure of cancer impact, YLLD, in individuals who die of their disease. YPLL and YLLD complement traditional indicators of mortality and help place CNS tumors in the context of other childhood malignancies.

## Introduction

Cancer in children and adolescents <20 years old is rare, but is one of the leading causes of death among children in the United States 1,2. Age-adjusted incidence rates (AAIRs) of cancer diagnosed in individuals <20 years old have increased significantly since 1975, and this increase has particularly been driven by increases in the incidence of brain and other central nervous system (CNS) tumors and leukemia 3. The most common cancers in children <20 years old in the United States are CNS tumors (AAIR = 5.26 per 100,000 population), leukemia (AAIR = 4.5 per 100,000 population), non-Hodgkin lymphoma (AAIR = 1.1 per 100,000 population), and Hodgkin lymphoma (AAIR = 1.2 per 100,000 population) 2,4.

Mortality rates of childhood cancer have decreased by over 50% between 1975 and 2014 3. Between 2003 and 2009, the 5-year survival rate for children <20 years old diagnosed with any kind of cancer was 83% 3. Survival rates vary significantly by tumor type. Improvements in survival after diagnosis with CNS tumors have not kept pace with more common cancers of childhood 3,5. While survival statistics provide important information for cancer surveillance, mortality rates cannot fully describe the burden caused by premature mortality due to childhood cancers. Previous large cohort studies—in particular, the Childhood Cancer Survivor Study 6—have provided description of the impact of childhood cancer on adult survivors. Few studies, however, have examined the impact of childhood death due to cancer. We sought to measure the impact of death due to childhood brain and other CNS tumors by examining *years of potential life lost* (YPLL), a critical benchmark for measuring both the public health and economic impact of diseases. We calculate YPLL of specific CNS tumor histologies and compare these results to other common childhood and adolescent cancers. Furthermore, we describe *years of life lived with disease* (YLLD) in children and adolescents who die of these tumors in order to better measure the impact of childhood cancer on individuals. These statistics are particularly useful in CNS tumors and other rare cancers where overall incidence and/or survival calculations fail to communicate the impact of these tumors in comparison to more common cancers.

## Materials and Methods

**Figure 1:**
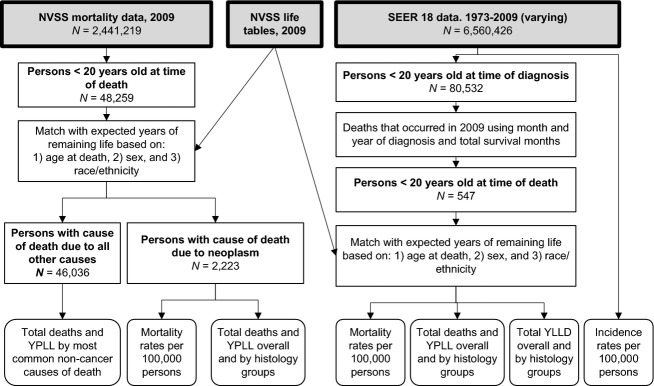
Overall study schematic of analytic pathway using data from the National Vital Statistics System (NVSS) and the Surveillance, Epidemiology and End Results (SEER) program to measure years of potential life lost (YPLL).

This analysis of population-based and de-identified datasets was conducted under a protocol deemed exempt by the University Hospitals Case Medical Center Institutional Review Board (IRB). The methods used in this analysis have been previously used to describe the burden of CNS tumors in the United States 7. This analysis utilized data from two sources in order to capture overall YPLL for the United States, as well as to assess YPLL and YLLD by specific histologies (Fig.1). Mortality data for the United States for the year 2009 was obtained from the National Vital Statistics System (NVSS) maintained by the Centers for Disease Control and Prevention's National Center for Health Statistics (NCHS) 8. This dataset includes all recorded death certificates in the United States in 2009. Cause of death is coded according to the International Statistical Classification of Diseases and Health Related Problems 10th revision (ICD-10). From this dataset, we selected all persons who were <20 years old at time of death in 2009. Life tables describing the future life expectancy for persons conditional on having survived to each year of age for the year 2009 were also obtained from NCHS 9. At the time this analysis was conducted, 2009 was the most recent year for which life tables were available, which resulted in the selection of this year for all other data sources. Each death record was matched with expected years of potential life based on age at death, gender, race, and ethnicity to estimate potential remaining years of life at time of death.

Causes of death in the NVSS dataset included all neoplasms (ICD-10 codes: C00-C97, D00-D48), brain and other CNS tumors (including both malignant and nonmalignant tumors: C70-C72, C75.1-C75.3, D32, D33, D35.2-D35.4, D42, D43.0-D43.2, D44.3-D44.5), leukemia (ICD-10: C91-C95), non-Hodgkin lymphoma (ICD-10: C82-C85, C96.3), and Hodgkin lymphoma (ICD-10: C81). YPLL was calculated as the difference between age of death and the average life expectancy for a person of the same age, race, and ethnicity in the United States in 2009. Total YPLL was the sum of YPLL among individuals who die of cancer, and mean YPLL was calculated by dividing total YPLL by the number of deaths. The NVSS dataset was also used to calculate mortality rates per 100,000 population for each histology group.

In order to assess histology-specific YPLL and YLLD, we utilized the Surveillance, Epidemiology and End Results (SEER) dataset released by the National Cancer Institute 10. This dataset contains all cancer diagnoses from within 18 SEER-funded registries (representing ∽26% of the United States population) between 1973–2000 (varying depending on registry) and 2009, as well as active follow-up for outcomes. Starting in 2004 with the passage of the Benign Brain Tumor Cancer Registries Amendment, the SEER registries also began collecting nonmalignant brain tumor cases. Using only persons who died as a result of their cancer, we used month of diagnosis, year of diagnosis, survival months, and age of diagnosis to calculate approximate month and year of death and approximate age at death. Each death record was matched with expected years of potential life based on age at death, gender, race, and ethnicity to estimate potential remaining years of life at time of death. Diagnoses in the SEER dataset included brain and other CNS tumors (International Classification of Diseases for Oncology, 3rd edition [ICD-O-3] site codes: C70-C72, C75.1-C75.3, C30.0 for ICD-O-3 histology codes 9522-9523 only) 2, leukemia (ICD-O-3 histology codes 9800-9949, excluding ICD-O-3 Site Codes C70-C72, C75.1-C75.3), non-Hodgkin lymphoma (ICD-O-3 histology codes 9590-9649, 9670-9729, excluding Site Codes C70-C73, C75.1-C75.3), and Hodgkin lymphoma (ICD-O-3 histology codes 9650-9669, excluding Site Codes C70-C72, C75.1-C75.3). Please see Table1 for the total number of deaths in 2009 due to each of these causes. Mortality and incidence rates per 100,000 population for each histology group were also calculated from the SEER dataset.

**Table 1:** Demographics for persons 0–19 years old who died of selected neoplastic histologies in 2009 (NVSS and SEER)

	All neoplasms		Brain and CNS neoplasms		Leukemia		Non-Hodgkin lymphoma		Hodgkin disease	
Data Source	NVSS	SEER	NVSS	SEER	NVSS	SEER	NVSS	SEER	NVSS	SEER
Total deaths	2223	547	678	172	572	134	77	20	22	6
Sex										
Male	1231 (55.4%)	288 (52.7%)	368 (54.3%)	91 (52.9%)	338 (59.1%)	75 (56.0%)	42 (54.5%)	9 (45.0%)	13 (59.1%)	4 (66.7%)
Female	992 (44.6%)	259 (47.3%)	310 (45.7%)	81 (47.1%)	234 (40.9%)	59 (44.0%)	35 (45.5%)	11 (55.0%)	9 (40.9%)	2 (33.3%)
Race/ethnicity										
Non-Hispanic white	1206 (54.3%)	228 (41.7%)	377 (55.6%)	81 (47.1%)	291 (50.9%)	39 (29.1%)	43 (55.8%)	10 (50.0%)	14 (63.6%)	1 (16.7%)
Non-Hispanic black	362 (16.3%)	78 (14.3%)	104 (15.3%)	24 (14.0%)	65 (11.4%)	14 (10.4%)	16 (20.8%)	3 (15.0%)	5 (22.7%)	3 (50.0%)
Hispanic white	506 (22.8%)	176 (32.2%)	150 (22.1%)	45 (26.2%)	176 (30.8%)	64 (47.8%)	14 (18.2%)	5 (25.0%)	2 (9.1%)	1 (16.7%)
Hispanic black	15 (0.7%)	9 (1.6%)	6 (0.9%)	2 (1.2%)	0 (0.0%)	4 (3.0%)	1 (1.3%)	0 (0.0%)	0 (0.0%)	0 (0.0%)
Hispanic other	9 (0.4%)	2 (0.4%)	3 (0.4%)	0 (0.0%)	4 (0.7%)	1 (0.7%)	1 (1.3%)	0 (0.0%)	0 (0.0%)	1 (16.7%)
American Indian/American Native	23 (1.0%)	4 (0.7%)	8 (1.2%)	2 (1.2%)	4 (0.7%)	2 (1.5%)	0 (0.0%)	0 (0.0%)	0 (0.0%)	0 (0.0%)
Asian Pacific Islander	102 (4.6%)	45 (8.2%)	30 (4.4%)	17 (9.9%)	32 (5.6%)	7 (5.2%)	2 (2.6%)	2 (10.0%)	1 (4.5%)	0 (0.0%)
Unknown	0 (0.0%)	5 (0.9%)	0 (0.0%)	1 (0.6%)	0 (0.0%)	3 (2.2%)	0 (0.0%)	0 (0.0%)	0 (0.0%)	0 (0.0%)
Age										
0–4	542 (24.4%)	111 (20.3%)	168 (24.8%)	36 (20.9%)	147 (25.7%)	30 (22.4%)	7 (9.1%)	3 (15.0%)	0 (0.0%)	0 (0.0%)
5–9	522 (23.5%)	143 (26.1%)	225 (33.2%)	71 (41.3%)	118 (20.6%)	24 (17.9%)	12 (15.6%)	3 (15.0%)	3 (13.6%)	1 (16.7%)
10–14	471 (21.2%)	124 (22.7%)	148 (21.8%)	37 (21.5%)	131 (22.9%)	39 (29.1%)	21 (27.3%)	5 (25.0%)	4 (18.2%)	1 (16.7%)
15–19	688 (30.9%)	169 (30.9%)	137 (20.2%)	28 (16.3%)	176 (30.8%)	41 (30.6%)	37 (48.1%)	9 (4.05%)	15 (68.2%)	4 (66.7%)

CNS, central nervous system; NVSS, National Vital Statistics System; SEER, Surveillance Epidemiology and End Results.

YLLD was defined as the difference between age at diagnosis and age of death for those who die of cancer before 20 years of age. Total YLLD was the sum of survival years for each person in the dataset, and mean YLLD was estimated by dividing total YLLD by number of deaths. All analyses were completed using R version 3.1.1 11 and SEER*Stat 8.1.5 12. All age-adjusted rates that were included have been adjusted to the 2000 U.S. standard population.

## Results

There were a total of 48,259 deaths in children <20 years old in the United States in 2009, of which 2223 were attributed to neoplasms (Table1). Within the SEER dataset, there were 547 deaths (∽25% of NVSS). These deaths more commonly occurred in males and white non-Hispanics. CNS tumors, leukemia, and lymphoma accounted for ∽61% of total deaths due to cancer in 2009. The largest proportion of deaths occurred in children ages 15–19 years, largely due to leukemia. For children who died at ages 0–4 years, CNS tumors (including tumors of the brain, spine, or other CNS locations) caused ∽31% of all deaths as compared to 43.1% of those in children ages 5–9 years (Table2). Mean YPLL were not significantly different between the NVSS and SEER populations (Table2).

**Table 2:** Total and mean YPLL (with median age at death) for persons 0–19 years old who died of selected neoplastic histologies in 2009 overall and by 5-year age at death groups

	NVSS										SEER									
	Overall (0–19)		0–4		5–9		10–14		15–19		Overall (0–19)		0–4		5–9		10–14		15–19	
	Total deaths	Total YPLL	Total deaths	Total YPLL	Total deaths	Total YPLL	Total deaths	Total YPLL	Total deaths	Total YPLL	Total deaths	Total YPLL	Total deaths	Total YPLL	Total deaths	Total YPLL	Total deaths	Total YPLL	Total deaths	Total YPLL
All neoplasms	2223	153,390.4	542	41,776.0	522	37,577.0	471	31,622.8	688	42,414.6	547	37,889.1	111	8609.0	143	10,376.7	124	8431.3	169	10,472.1
Brain and CNS neoplasms	678	47,631.5	168	13,005.0	225	16,229.7	148	9930.4	137	8466.4	172	12,190.0	36	2780.9	71	5169.6	37	2504.8	28	1734.7
Brain	626	43,854.7	149	11,542.1	216	15,580.6	140	9397.5	131	8087.5	154	10,912.7	30	2316.9	66	4808.3	33	2229.4	25	1558.1
Other CNS	42	3023.8	19	1462.9	9	649.1	8	532.9	6	378.9	18	1277.3	6	464.0	5	361.3	4	275.4	3	176.6
Leukemia	572	39,569.9	147	11,372.9	118	8501.7	131	8791.9	176	10,903.4	134	9323.1	30	2349.5	24	1748.7	39	2655.8	41	2569.1
Lymphocytic leukemia	255	17,594.9	47	3640.3	65	4691.9	74	4996.8	69	4265.9	67	4631.8	9	715.8	14	1021.6	25	1702.6	19	1191.8
Myeloid leukemia	204	14,154.1	66	5086.6	32	2308.2	36	2407.3	70	4352.0	61	4261.8	19	1481.6	7	505.1	14	953.2	21	1321.9
Non-Hodgkin lymphoma	77	5055.1	7	537.3	12	867.2	21	1401.8	37	2248.8	20	1344.5	3	226.9	3	221.7	5	329.9	9	566.0
Hodgkin lymphoma	22	1382.7	—	—	3	205.1	4	262.4	15	915.2	6	373.8	—	—	—	—	—	—	4	236.8

CNS, central nervous system; —, excluded due to low numbers; YPLL, years of potential life lost; NVSS, National Vital Statistics System; SEER, Surveillance Epidemiology and End Results.

**Figure 2:**
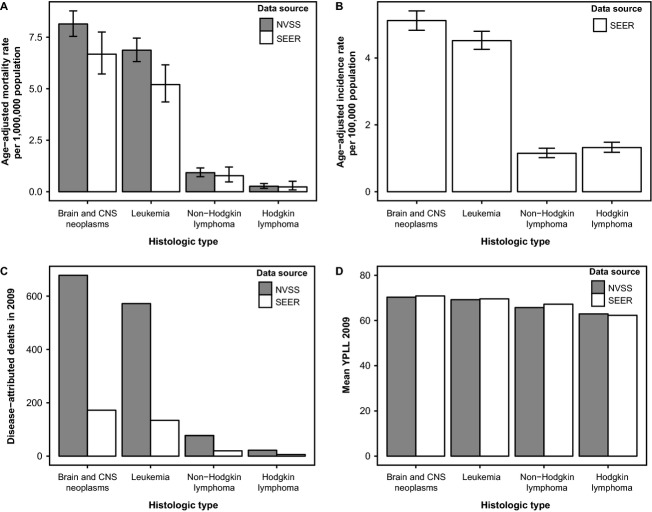
(A) Age-adjusted mortality rates and (B) age-adjusted incidence per 100,000 population for selected histologies, (C) total disease attributed deaths for selected histologies, and (D) mean years of productive life lost (YPLL) for persons 0–19 years old in 2009 for selected histologies (National Vital Statistics System [NVSS] and Surveillance, Epidemiology and End Results [SEER]).

The age-adjusted mortality rates for each of the four disease groups (CNS tumors, leukemia, Hodgkin lymphoma, non-Hodgkin lymphoma) were similar in both datasets in 2009 (Fig.2A) with CNS tumors having the highest age-adjusted mortality rate (NVSS: CNS = 8.1 per 1,000,000; leukemia = 6.9 per 1,000,000; Hodgkin lymphoma = 0.3 per 1,000,000; non-Hodgkin lymphoma = 0.9 per 1,000,000). CNS tumors also had the highest AAIR in 2009 (AAIR = 5.2, 95% CI = 4.8–5.4), with leukemia having the second highest AAIR (AAIR = 4.5, 95% CI = 4.3–4.8) (Fig.2B). In both datasets, approximately 30% of deaths were attributed to CNS tumors, ∽25% to leukemia, ∽3% to non-Hodgkin lymphoma, and ∽1% due to Hodgkin lymphoma (Fig.2C). There were a total of 153,390.4 YPLL due to neoplasms in 2009 (Table2), representing 4.4% of a total 3,489,798.0 YPLL due to all causes in individuals <20. Of these, ∽31% were due to CNS tumors, ∽26% to leukemia, ∽3% to non-Hodgkin lymphoma, and <1% to Hodgkin lymphoma. For each death due to disease, mean YPLL was 70.3 for CNS tumors, 69.2 for leukemia, 65.7 for non-Hodgkin lymphoma, and 62.9 for Hodgkin lymphoma (Fig.2D).

**Figure 3:**
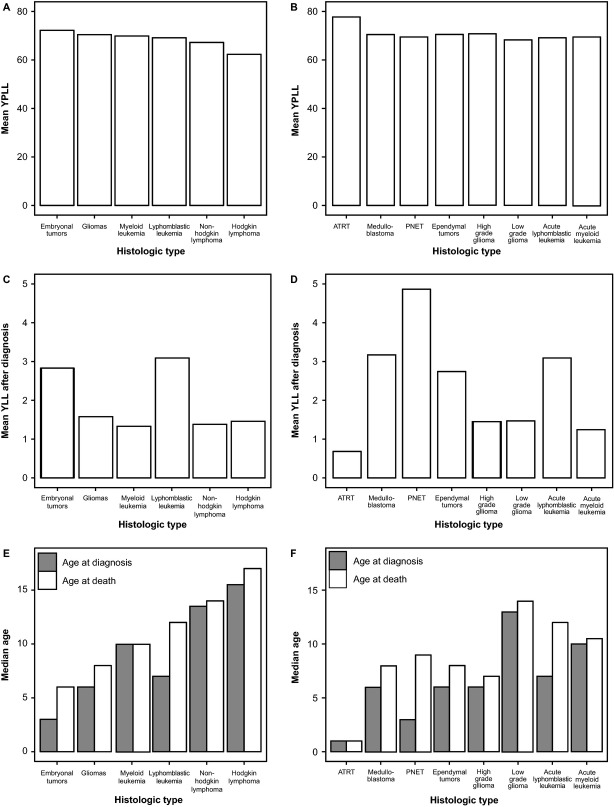
(A) Mean years of potential life lost (YPLL) due to selected tumor categories for persons 0–19 years old, (B) mean YPLL for selected central nervous system (CNS) tumor and leukemia histologies, (C) mean years of life lived with disease (YLLD) prior to death for persons 0–19 years old by selected tumor categories, (D) mean YLLD for selected CNS and leukemia histologies, (E) median age at diagnosis death by selected histologies, and (F) median age at diagnosis and death by selected CNS and leukemia histologies (SEER).

CNS tumors caused the highest loss of potential life years, followed by leukemia, non-Hodgkin lymphoma, and Hodgkin lymphoma (Fig.3A). Among all specific histologies examined, the histologic types with the highest mean YPLL were atypical teratoid/rhabdoid tumors (ATRT, ICD-O-3 histology code: 9508) (mean YPLL = 78.04) and high-grade gliomas (ICD-O-3 histology codes: 9381, 9401, 9440-9442, 9451, 9460 for all sites, 9380 and 9400 only for site code C71.7) (mean YPLL = 70.67) (Fig.3B). Mean YLLD was not significantly different between CNS tumors and other common childhood cancers in children and adolescents who die of cancer before 20 years of age (leukemia *P* = 0.224, non-Hodgkin lymphoma *P* = 0.308, and Hodgkin lymphoma *P* = 0.623) (Table3). The histologies with the highest mean YLLD were primitive neuroectodermal tumor (PNET) (mean YLLD = 4.59), medulloblastoma (mean YLLD = 3.17), and acute lymphoblastic leukemia (mean YLLD = 3.09). The histology with the lowest mean YLLD was ATRT (mean YLLD = 0.63). Individuals who died of gliomas, non-Hodgkin lymphoma, Hodgkin lymphoma, and myeloid leukemia lived the shortest after diagnosis on average (Fig.3C). There was a significant difference between the different embryonal subtypes: PNET had the longest mean YLLD, while ATRT had the shortest followed by acute myeloid leukemia (Fig.3D). Those diagnosed with gliomas and embryonal tumors died at the youngest median ages, while those diagnosed with non-Hodgkin lymphoma or Hodgkin lymphoma died at the oldest median ages (Fig.3E). Of all embryonal tumors, ATRT had both the lowest median age of diagnosis and death (Fig.3F).

**Table 3:** Mean and total YPLL (with median age at death) and mean YLLD in 2009 (SEER)

Site or histologic grouping	Total deaths	Total YLLD	Total YPLL	Median age at diagnosis	Median age at death
All neoplasms	547	1088.2	37,889.1	8.0	10.0
Brain and CNS neoplasms	172	326.3	12,190.0	6.0	8.0
Brain	154	293.3	10,912.7	6.0	8.0
Glioma	104	163.8	7327.8	6.0	8.0
Low-grade glioma	11	16.2	750.9	13.0	14.0
High-grade glioma	77	111.3	5441.6	6.0	7.0
Ependymal tumors	11	30.2	775.7	6.0	8.0
Embryonal tumors	43	121.8	3095.8	4.0	8.0
Atypical teratoid/rhabdoid tumor	10	41.3	780.4	1.0	1.0
Primitive neuroectodermal tumor	9	53.5	624.7	4.0	11.0
Medulloblastoma	23	72.9	1620.8	6.0	8.0
Other CNS	18	33.0	1277.3	5.0	8.0
Leukemia	134	294.7	9323.1	8.5	11.0
Lymphoid leukemia	67	207.2	4631.8	7.0	12.0
Acute lymphoblastic leukemia	67	207.2	4631.8	7.0	12.0
Myeloid leukemia	61	81.3	4261.8	10.0	10.0
Acute myeloid leukemia	46	57.2	3202.9	10.0	10.5
Non-Hodgkin lymphoma	20	27.6	1344.5	13.5	14.0
Hodgkin lymphoma	6	8.8	373.8	15.5	17.0

CNS, central nervous system; SEER, Surveillance Epidemiology and End Results; YLLD, years of life lived with disease; YPLL, years of potential life lost.

**Figure 4:**
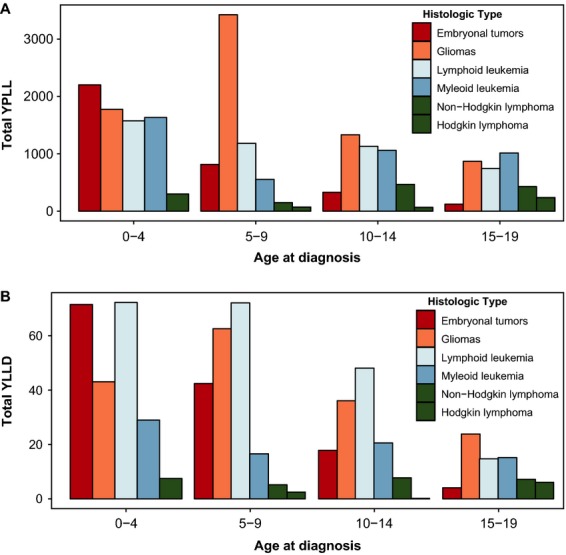
Total (A) years of potential life lost (YPLL) and (B) total years of life lived with disease (YLLD) for persons 0–19 years old who died of selected histologic groups by age of diagnosis (SEER).

Both YPLL and YLLD varied with age at diagnosis for all tumor types (Table4). For children diagnosed with cancer between 0 and 4 years, embryonal tumors were the largest cause of YPLL (Fig.4A). For children 5–14, gliomas were the largest contributor to YPLL with myeloid leukemia causing the highest YPLL in those diagnosed at ages 15–19. Embryonal tumors and acute lymphoblastic leukemia caused nearly equal amounts of YLLD in children ≤4 years at time of diagnosis (Fig.4B). In children 5–14 years, lymphoid leukemia was the biggest source of YLLD. Once children were between 15 and 19 years, gliomas were the largest cause of YLLD with almost no YLLD due to embryonal tumors.

**Table 4:** Total YPLL and YLLD for persons 0–19 years old who died of selected histologies by 5-year age of diagnosis groups in 2009 (SEER)

	0–4			5–9			10–14			15–19		
Histologic grouping	Total deaths	Total YLLD	Total YPLL	Total deaths	Total YLLD	Total YPLL	Total deaths	Total YLLD	Total YPLL	Total deaths	Total YLLD	Total YPLL
Brain and CNS neoplasms	56	119.7	4210.1	67	117.4	4824.7	31	60.4	2047.3	28	78.2	1107.9
All brain	30	18.6	2316.9	66	107.9	4808.3	33	91.3	2229.4	25	75.6	1558.1
Glioma	24	43.1	1775.4	47	62.6	3424.8	20	36.1	1331.2	14	23.8	867.8
Low-grade glioma	—	—	—	—	—	—	4	8.7	259.3	3	4.6	193.7
High-grade glioma	18	32.9	1333.8	38	44.9	2762.0	13	18.1	877.3	9	17.2	540.2
Embryonal tumors	29	71.5	2201.3	12	42.4	814.5	5	17.8	328.8	—	—	—
Atypical teratoid/rhabdoid tumor	11	7.2	860.1	—	—	—	—	—	—	—	—	—
Primitive neuroectodermal tumor	8	49.1	572.8	—	—	—	—	—	—	—	—	—
Medulloblastoma	9	15.2	690.2	10	40.3	672.5	4	17.5	258.1	—	—	—
Leukemia	44	30.4	3360.2	28	84.8	1958.3	33	62.8	2190.3	29	116.8	1814.3
Lymphoid leukemia	21	72.3	1574.5	17	72.1	1182.5	17	48.1	1130.6	12	14.8	744.2
Acute lymphoblastic leukemia	21	72.3	1574.5	17	72.1	1182.5	17	48.1	1130.6	12	14.8	744.2
Myeloid leukemia	21	29.0	1633.6	8	16.6	553.8	16	20.6	1059.7	16	15.2	1014.7
Acute myeloid leukemia	16	22.8	1254.5	5	5.5	345.4	12	19.4	783.6	13	9.5	819.4
Non-Hodgkin lymphoma	4	7.5	300.1	—	—	—	7	7.8	467.1	7	7.2	428.8
Hodgkin lymphoma	—	—	—	—	—	—	—	—	—	4	6.1	236.8

CNS, central nervous system; SEER, Surveillance Epidemiology and End Results; —, excluded due to low numbers; YLLD, years of life lived with disease; YPLL, years of potential life lost.

## Discussion

CNS tumors are the second most common malignancy in children and are the most common cause of cancer-related death in 0–19 year olds 13. However, these tumors often fail to receive the attention or research funding commensurate with their impact 14. We sought to examine YPLL to complement and counterbalance measures of impact based only on incidence and total mortality. YPLL measures the total number of years cut short due to disease and, therefore, gives a more comprehensive assessment of diseases that kill early or are incurable 15. The second instrument used in this report, YLLD, measures the total years from diagnosis to death for those who die of malignancy before 20 years of age. YLLD presents another method to measure the impact of disease and may provide a potential benchmark to gauge clinical success by using registry data for tumors that are rapidly fatal.

Prior reports have examined YPLL due to cancer in the general population 16 and among childhood CNS tumors by site of tumor 7. Similar to our results, Thuppal et al. found 697 total deaths due to brain and CNS tumors in children 0–19 in 2001, compared to the 678 total deaths due to brain and CNS tumors in children 0–19 found in this analysis. Unlike prior reports, our study is the first description of YPLL in childhood tumors defined by histological subtype. An understanding of survival statistics by histological subtype is essential to the analysis of childhood CNS tumors, which otherwise represent a diverse population of tumors with widely variable outcomes. An analysis by histological subtype is made possible by combining two large national registry datasets, SEER and NVSS. Although these two data sources cover different and overlapping geographic areas, a comparison of relative age-adjusted mortality, disease-attributed death, and mean YPLL in 2009, as well as population demographics, demonstrates concordant results and confirms the validity of the SEER sample. In addition, SEER incidence data in 2009 and median age at diagnosis reported here remain remarkably similar to data presented from the North American Association of Central Cancer Registries covering 46 states from 2004 to 2007 17, further validating the SEER sample as representative of the United States.

Our analysis demonstrates that CNS tumors are responsible for the greatest loss of total potential life years in children 0–19 years old in the United States, compared to childhood leukemia and lymphoma (47,631.5 years [31%] vs. 39,569.9 [26%] and 11,716.8 [7.6%], respectively). After considering specific histology classifications, gliomas (including high-grade glioma, low-grade glioma, and ependymoma) are responsible for the greatest loss of potential life years in children and adolescents—almost 60% greater than acute lymphoblastic leukemia. Among gliomas, high-grade glioma is responsible for approximately 75% of YPLL. Although high-grade gliomas are uncommon compared to acute lymphoblastic leukemia, this analysis reflects the loss of life due to this disease and suggests the potential for future clinical improvement. Furthermore, YPLL measures the impact of disease on society and complements crude mortality indicators by demonstrating the importance of childhood brain tumors to overall life lost due to cancer. Childhood brain and CNS tumors are the source of 31.1% of all YPLL due to childhood cancer, and the source of 1.4% of all YPLL due to all causes in 2009. In comparison, the causes of death that contributed the largest amount of YPLL were death due to perinatal causes (1,015,428.7 YPLL, or 29.1%), accidents (692,637.5 YPLL, or 20.0%), and congenital causes (494,527.9 YPLL or 14.2%).

Mean YPLL offers another view of the impact of cancer-related deaths by measuring the number of years of life lost due to an individual cancer death. In this measure, as well, the impact of brain and other CNS tumors is greater than leukemia or lymphoma in children <20 years old and reflects the young age at which death occurs in these children. YPLL has been used to measure predicted clinical impact of new therapeutic modalities 18, and mean YPLL is considered an important factor in the allocation of research funds 14. For childhood CNS tumors, these measures demonstrate the relative impact of these tumors on society.

YLLD is a measure of cancer's impact on children and their families, reflecting the time spent between diagnosis and death for those who eventually die of their cancer. While it is impossible to predict the quality of life or extent of disability during this period, YLLD represents the area under the survival curve for the portion of individuals who die of their disease and presents another measure of clinical progress for tumors that are fatal. YLLD can be affected by available therapeutic options as well as disease severity. Total YLLD due to brain and other CNS tumors (gliomas and embryonal tumors) outpaces that due to leukemia (lymphoid and myeloid) in children <10 years old, although acute lymphoblastic leukemia remains the single largest contributor to YLLD in this age group. In our study, mean YLLD was greatest among children and adolescents with medulloblastoma, PNET, and acute lymphoblastic leukemia, which may reflect longer treatment courses or multiple treatment options that may prolong survival in these specific diseases. Mean YLLD was smallest among individuals with ATRT (0.63 years) and those with high-grade glioma (including diffuse intrinsic pontine glioma) between 5 and 9 years old (1.18 years), reflecting the shortened life expectancies for these diagnoses and the paucity of effective therapies for relapsed/refractory disease. To use YLLD as a potential benchmark of clinical response, more research is needed. Future studies should investigate the effect of treatments and demographic factors on YLLD to validate this measure. In addition, the end of life is a highly individual time during which some families may wish to extend life and others may focus on treating symptoms. Measuring quality of life for patients and their families during this period will be essential to understand the impact and importance of YLLD.

Our study is subject to certain limitations. While registry data from large national databases (SEER and NVSS) offer the advantage of covering a substantial proportion of the United States population, we are limited to the data captured in these registries. The effect of therapies and the quality of life during treatment are not available in these data and should be investigated using different data sources. Our analysis also investigates a single time point (the year 2009) as characteristic of the current impact of death due to childhood cancer. Statistics on death certificates often lag behind the present day; 2009 is the most current year for which life expectancy tables are available from the NCHS. In general, 2009 appears to be representative of recent epidemiology studies of pediatric and adolescent cancers, although the incidence of new CNS tumors exceeded leukemia in that year. Because this analysis focuses on deaths occurring in 2009, rather than new cancer incidence, CNS tumor incidence will not affect YPLL or YLLD statistics reported here.

While our analytic methods focus on childhood deaths due to CNS tumors, we may fail to capture deaths that occur after 20 years of age or in long-term survivors of cancer 19. This may result in the under-counting of both YPLL and YLLD for pediatric cancer.

Although CNS tumors were first recorded in the SEER database in 1973, SEER sites were added until the year 2000, and nonmalignant CNS tumors began to be included starting 1 January 2004 20. This may limit the YPLL and YLLD in SEER sites added recently and will decrease the apparent impact of histologically benign tumors, where each subject could only contribute a maximum of 5 years to either analysis. This may contribute to the interpretation of YLLD for low-grade glioma which is only nominally greater than that for high-grade glioma (1.47 vs. 1.45 years) in our analysis. However, the diminished YLLD for low-grade glioma also reflects that perioperative and early mortality may be a significant factor affecting overall mortality for some low-grade gliomas.

## Conclusion

CNS tumors are the second most common malignancy in children but have the highest cost in YPLL. Among specific histologies, high-grade gliomas—including diffuse intrinsic pontine glioma—have the highest YPLL, almost 17% greater than the malignancy responsible for the next greatest YPLL, acute lymphoblastic leukemia. ATRT is the tumor responsible for the shortest mean YLLD, reflecting its rapid progression from diagnosis to death. This study reports on measures of cancer impact that are complementary to traditional indicators such as total mortality, and places pediatric and adolescent CNS tumors in the context of other common pediatric and adolescent malignancies. The epidemiologic data contained in this report contribute to expanding our understanding of the far-reaching implications of CNS tumors on the pediatric and adolescent population in the United States and, hopefully, may stimulate research into reducing the impact of these devastating tumors.
